# Sarcomatoid Carcinoma of the Small Intestine: A Case Report

**DOI:** 10.7759/cureus.68850

**Published:** 2024-09-07

**Authors:** Umair Saleem, Tayyaba Ansari

**Affiliations:** 1 Medical Oncology, Peterborough City Hospital, Peterborough, GBR

**Keywords:** duodenal cancer, gastro-intestinal neoplasm, rare cancers, sarcomatoid carcinoma, sarcomatoid carcinoma of duodenum, upper gastro-intestinal bleed, upper gi cancer

## Abstract

Sarcomatoid carcinoma of the gastrointestinal tract is an extremely rare and aggressive tumor with both epithelial and mesenchymal characteristics, and it typically has a poor prognosis. We report the case of a 74-year-old male diagnosed with sarcomatoid carcinoma of the duodenum. The patient presented with gastrointestinal bleeding and was found to have a vascular tumor in the third part of the duodenum. Initial duodenal biopsies, repeat biopsies, and extensive immunohistochemical analysis confirmed a diagnosis of sarcomatoid carcinoma. Despite radical surgery and multiple lines of chemotherapy, including carboplatin and paclitaxel, the disease demonstrated aggressive progression, ultimately leading to the patient’s death two years post-diagnosis. This report highlights the challenges in diagnosing and treating sarcomatoid carcinoma of the small intestine, the limited efficacy of current therapeutic options, and the need for further research to establish effective treatment protocols.

## Introduction

Sarcomatoid carcinoma is an extremely rare tumor in the gastrointestinal tract and is associated with a poor prognosis [1]. This carcinoma exhibits both epithelial and mesenchymal characteristics. Sarcomatoid carcinomas have been reported in various locations, including the gallbladder [2], stomach [3], esophagus [4], and colon [5], but they are rarely found in the small intestine [6-8]. To date, only 32 cases of sarcomatoid carcinoma of the small bowel have been documented in the literature [9]. This type of carcinoma is highly aggressive, with a median survival of only a few months and an overall five-year survival rate of approximately 20% [10]. We present the case of a 74-year-old male diagnosed with sarcomatoid carcinoma of the duodenum, which demonstrated highly aggressive behavior.

## Case presentation

**Figure 1:**
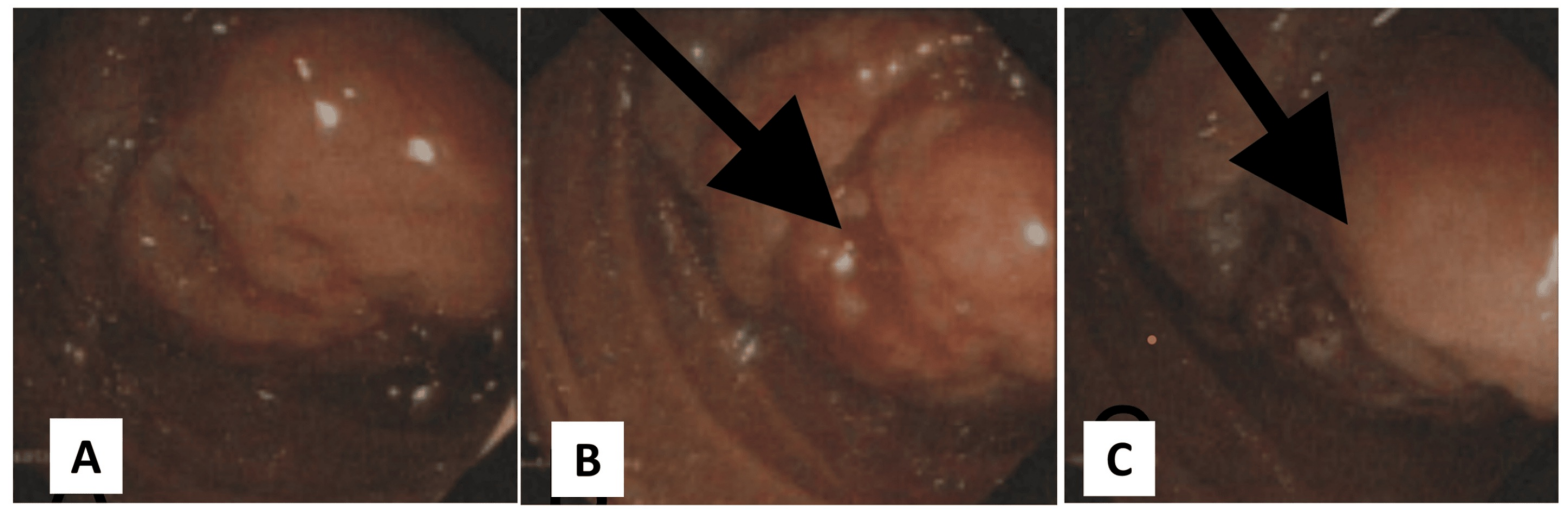
(A) Esophagogastroduodenoscopy showing vascular tumor with erosion in third part of the duodenum. (B) Arrow indicating erosion. (C) Arrow indicating mass

**Figure 2:**
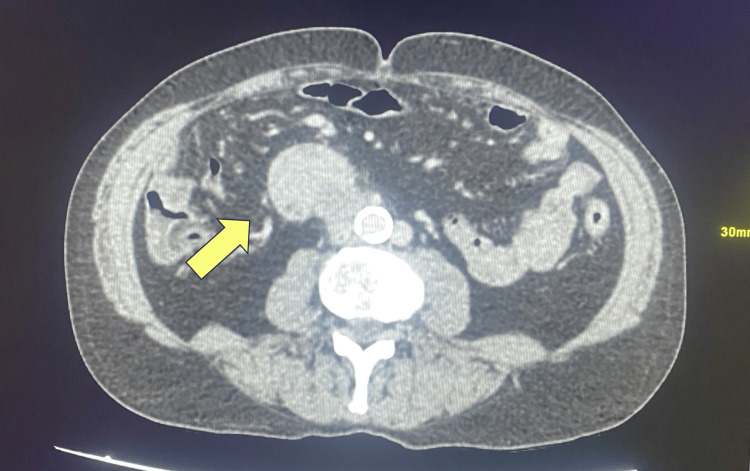
Axial CT scan showing mass lesion arising from the third part of the duodenum CT: computed tomography

**Figure 3:**
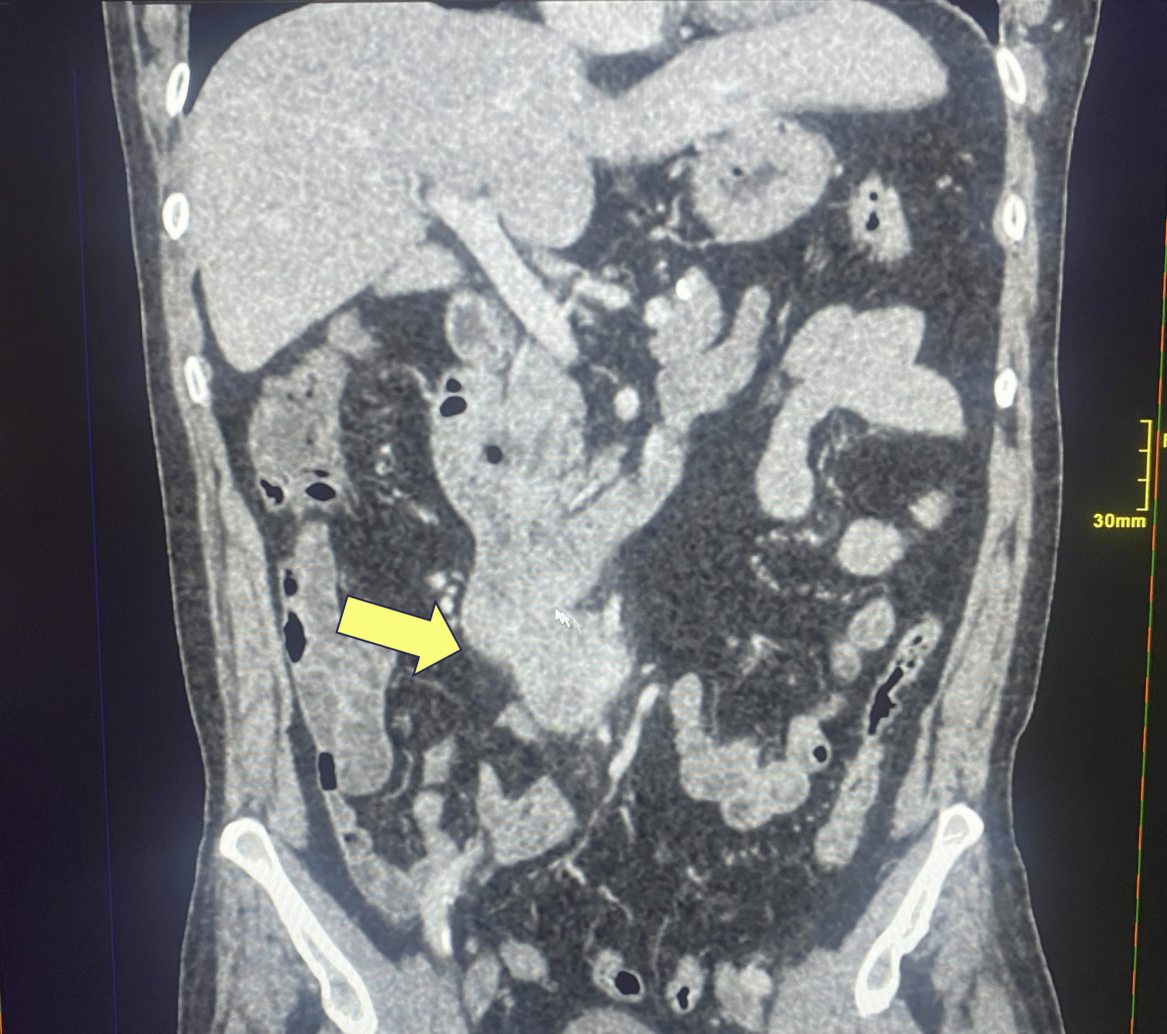
Coronal CT scan showing mass lesion arising from the third part of the duodenum CT: computed tomography

A 74-year-old male with a history of hypertension, ischemic heart disease, asthma, coronary artery bypass graft surgery, and gout was admitted to Peterborough City Hospital after experiencing melena for two weeks. On physical examination, he appeared pale and had a postural drop in blood pressure. His abdomen was soft and non-tender. Blood tests revealed significant anemia, with hemoglobin of 75 g/L; the remaining blood results were unremarkable. Esophagogastroduodenoscopy revealed a vascular tumor in the third part of the duodenum, occupying most of the lumen, with initial bleeding that later resolved (Figure 1). CT of the neck, thorax, abdomen, and pelvis showed a mass lesion from the thickened third part of the duodenum, with no evidence of distant metastatic disease (Figures 2-3).

Duodenal biopsies suggested a high-grade sarcomatoid neoplasm involving the third part of the duodenum (D3). A second opinion from the team at Addenbrooke’s Hospital was sought and they recommended an urgent repeat biopsy, which was performed. Repeat biopsies revealed an infiltrative, highly pleomorphic tumor composed of epithelioid and spindle cells with bizarre multinucleated giant tumor cells in a desmoplastic stroma. Samples sent to University College London confirmed an undifferentiated pleomorphic neoplasm. The molecular analysis identified significant genetic alterations in key cancer-related genes, disrupting several tumor suppressor functions. Notably, there was a loss of CDKN2A, CDKN2B, and MTAP, along with an MTAP truncation. Additionally, the presence of a TP53 R282W mutation indicated impaired regulation of cell growth, while PIK3CA H1047L and PTEN R233* mutations suggested activation of oncogenic pathways linked to cell survival and proliferation. A DNMT3A R635W mutation was also detected, which is associated with various cancer-related processes.

Biomarker analysis showed a relatively low tumor mutation burden (TMB) of 2.52 mutations per megabase, and the tumor was microsatellite stable (MS-Stable), indicating a lower likelihood of response to immunotherapy. Variants of unknown significance (VUS) were also identified, including a TMPRSS2-TMPRSS2 rearrangement and a D400N alteration in HDAC1. Immunohistochemistry was negative for markers including MNF116, S100, HMB-45, Melan-A, smooth muscle actin, desmin, myogenin, and CD34. There was non-specific cytoplasmic staining for MyoD1. Further testing at the Royal National Orthopaedic Hospital revealed numerous scattered tumor cells expressing epithelial membrane antigen (EMA) and p63, while DOG1 and ERG were negative. The presence of p63 expression, which is uncommon in soft tissue sarcomas, along with the site and EMA expression, supported a diagnosis of sarcomatoid carcinoma with loss of cytokeratin expression rather than a sarcoma.

The hepatobiliary pancreatic specialist multidisciplinary team at Peterborough City Hospital and Leicester recommended resection. The patient underwent duodenal and caval resection at Leicester with some vascular reconstruction. The resected specimen confirmed a high-grade tumor consistent with sarcomatoid carcinoma and the edges of the specimen were free of cancer cells, indicating that the cancer was completely excised. A surveillance CT scan at six months revealed an ill-defined mass extending anterior to the inferior vena cava and possibly involving bowel loops, indicating recurrence. Positron emission tomography showed increased metabolic activity along the right psoas muscle and multiple metabolically active lymph nodes in the lower para-aortic, right iliac, and right pelvic sidewall regions, measuring up to 36 mm with a standardized uptake value of 11.2.

Chemotherapy with capecitabine and oxaliplatin was initiated for the recurrence based on recommendations from Cambridge University Hospital, as no suitable clinical trials were available then. A CT scan after four cycles of chemotherapy showed progressive disease. Second-line treatment with carboplatin and paclitaxel was started while awaiting approval for individual funding for everolimus. The Royal Marsden suggested exploring everolimus, an mTOR inhibitor, due to the identification of a PIK3 mutation. However, the funding request was declined, as the treatment was considered experimental. A follow-up CT scan after four cycles of carboplatin and paclitaxel showed stable disease, which was the first slightly reassuring result. The patient completed six cycles of chemotherapy, which stabilized the disease and caused a minimal reduction in the size of the retroperitoneal and pelvic nodal disease, as evidenced by the CT scan.

Unfortunately, a subsequent CT scan showed disease progression, with an increase in the size of the duodenal mass leading to increased duodenal dilatation. It also revealed a large abdominopelvic mass arising from the medial aspect of the right psoas muscle, which had increased in size compared to previous scans. Additionally, metastatic pulmonary nodules confirmed further disease progression. Shortly after the scan, the patient was admitted to the hospital due to general deterioration and confusion. Brain imaging ruled out metastatic lesions, and he was treated for hypercalcemia and a presumed infection. He was fast-tracked for discharge and died peacefully at home a few days later.

## Discussion

Sarcomatoid carcinoma is a rare and controversial tumor that exhibits both carcinomatous and sarcomatous features. It is known by various names, including carcinosarcoma, metaplastic carcinoma, spindle cell carcinoma, and pleomorphic carcinoma [1]. Sarcomatoid carcinoma of the small intestine predominantly affects elderly patients, with a mean age of presentation of 57 years [1]. Common presenting symptoms include abdominal pain, intestinal obstruction, palpable abdominal mass, gastrointestinal bleeding, and anemia. In this case, the patient presented with signs of gastrointestinal bleeding. The diagnosis of sarcomatoid carcinoma is challenging due to its overlapping histopathological features with other spindle cell tumors. A thorough understanding of its clinicopathologic characteristics is required for accurate diagnosis and appropriate clinical management.

Histologically, sarcomatoid carcinoma may present with either a biphasic or monophasic pattern [1]. The typical biphasic pattern consists of a mixture of epithelial-like and mesenchymal-like cells, while monophasic tumors are characterized by a predominance of mesenchymal-like components with minimal to absent epithelioid areas [1]. In our case, the tumor displayed a highly pleomorphic pattern, comprising epithelioid and spindle cells with bizarre multinucleated giant tumor cells in a desmoplastic stroma. A broad panel of immunohistochemical markers was used to narrow down the differential diagnosis, which included epithelial origin cancers (MNF116), melanoma (S100, HMB-45, Melan-A), and rhabdomyosarcoma (myogenin). The absence of desmin also ruled out leiomyosarcoma and negative DOG1 staining excluded gastrointestinal stromal tumor.

Surgical resection is the primary treatment for solid tumors and remains the most effective option for sarcomatoid carcinoma of the small intestine [1,7]. Neither radiotherapy nor chemotherapy has been shown to improve survival rates [1] significantly. While the average survival duration is generally only a few months, this patient survived for approximately two years post-diagnosis, representing one of the longer survival periods reported.

Currently, there are no standardized guidelines for adjuvant treatment with chemotherapy or radiotherapy in sarcomatoid carcinoma. Our patient underwent radical surgery followed by chemotherapy. Due to the tumor’s aggressive biological behavior, he was kept on short-term follow-up. Disease progression continued in subsequent scans following surgery and first-line adjuvant chemotherapy. However, second-line chemotherapy with carboplatin and paclitaxel stabilized the disease for six months and led to minimal tumor regression. Unfortunately, a follow-up scan two months later revealed further progression, which was accompanied by rapid clinical deterioration and death within a few weeks.

## Conclusions

Sarcomatoid carcinoma of the intestine is an extremely aggressive cancer with a significantly poor prognosis. Due to its rarity and complexity, the diagnosis and treatment are challenging, and no established treatment guidelines are currently available. This report highlights the immunohistochemical features of sarcomatoid carcinoma and its short-term response to second-line adjuvant/palliative chemotherapy with carboplatin and paclitaxel. Despite continuous treatment, the outcome was poor, with minimal impact on overall disease progression. Further research is needed to identify the best clinical approach for diagnosing and treating sarcomatoid carcinoma.
